# Identification of specific metabolic capacities associated with major extraintestinal pathogenic *Escherichia coli* lineages

**DOI:** 10.1128/jb.00421-25

**Published:** 2026-02-27

**Authors:** Guilhem Royer, Françoise Chau, David Vallenet, Erick Denamur, Colibafi and Septicoli groups, Colocoli group, Coliville group

**Affiliations:** 1 Unité de Bactériologie, Département de Prévention, Diagnostic et Traitement des Infections, AP-HP, Hôpital Henri Mondor 378967 , Créteil, France; 2 Université Paris Cité, INSERM, IAME 555089, Paris, France; 3 INSERM U955, Institut Mondor de Recherche Biomédicale 165200, Créteil, France; 4 LABGeM, Génomique Métabolique, Genoscope, Institut François Jacob, CEA, CNRS, Université Evry, Université Paris-Saclay 27048, Evry, France; 5 AP-HP, Laboratoire de Génétique Moléculaire, Hôpital Bichat, Paris, France; University of Virginia School of Medicine, Charlottesville, Virginia, USA

**Keywords:** niche differentiation, metabolism, *Escherichia coli*, pangenome

## Abstract

Bacterial niche colonization relies on multiple factors, among which the metabolic capacity to utilize specific substrates is pivotal. As a gut commensal of humans and other vertebrates acting as an intestinal and extraintestinal opportunistic pathogen, *Escherichia coli* faces such environmental pressures. We therefore aimed to identify (i) metabolic patterns associated with *E. coli* lifestyle (commensal versus extraintestinal pathogenic *E. coli* [ExPEC]) and (ii) ExPEC-specific metabolic features that could help explain the emergence and success of major pandemic clones. Using a pangenomic framework coupled with metabolic pathway prediction, we analyzed 1,498 well-characterized *E. coli* strains collected over 17 years in France, including adult commensal strains (*n* = 370) and ExPEC strains involved in severe infections (bloodstream infections from various portals of entry and pneumonia) (*n* = 1,128). Although metabolism was more conserved than gene content, substantial metabolic diversity was observed, with over 50% pathways being variable, mainly involving biosynthetic and degradation processes. No pathway was specifically associated with lifestyle; metabolic profiles primarily reflected phylogeny. However, several clone-specific metabolic capacities were identified. Some may support extraintestinal survival during infection, such as the 5’-deoxynucleoside recycling pathway enriched in the major ExPEC clone STc69 from phylogroup D. Conversely, in phylogroup B2, clone-specific pathways enabled the degradation of plant-derived compounds, potentially facilitating gut colonization through niche-defining nutrients. Notably, D-apiose degradation pathway analysis revealed a functional pathway strongly associated with the pandemic clones STc131 and STc14. Overall, these lineage-specific metabolic capacities may contribute to the ecological success and dissemination of dominant ExPEC clones.

**IMPORTANCE:**

According to the nutrient-niche hypothesis, bacteria must exploit distinct substrates to grow and persist in their various habitats. Such niche differentiation is at play among the commensal and pathogenic *E. coli* populations. With this in mind, we search for specific associations between metabolic pathways and strain origin (commensal versus severe extraintestinal infections). Metabolic profiles were predominantly shaped by phylogeny, reflecting the species’ clonal structure and the close link between phylogenetic background and lifestyle. Among the lineage-specific determinants, we identified several pathways associated with worldwide spread clones responsible for bloodstream infections, supporting the existence of clone-specific strategies for niche adaptation.

## INTRODUCTION

Metabolism is key to bacterial adaptation to nutritional environments. This has been extensively studied in *Escherichia coli*, a species that includes both commensal, intestinal (intestinal pathogenic *E. coli* [InPEC]) and extraintestinal pathogenic (extraintestinal pathogenic *E. coli* [ExPEC]) strains (1, 2). Although *E. coli* primarily inhabits the intestinal tract of mammals and many other vertebrates (1, 3), it is also an opportunistic pathogen, using this habitat as a reservoir (2). Within the gut, the nutrient-niche hypothesis proposes that *E. coli* strains can coexist with resident microbiota by exploiting distinct nutrients (4). According to this theory, *n* populations may coexist if *n* distinct substrates are available, with population sizes controlled by substrate concentrations. However, not only nutrient availability but also strain-specific affinity for given nutrients contributes to colonization success and coexistence (5, 6). Spatial variation within the gut may add a third layer of complexity, whereby local microenvironments influence strain distribution (5, 7). Importantly, the nutrient-niche hypothesis extends beyond the intestinal tract and applies to other environments encountered during infection. For example, the survival and growth of uropathogenic *E. coli* in urine is facilitated by the *dsdCXA* locus, which enables D-serine utilization, an amino acid abundant in urine (8, 9).

*E. coli* exhibits a clonal population structure with eight major phylogroups, namely A, B1, B2, C, D, E, F, and G (1, 2, 10), as well as cryptic clades (I–V) that are phenotypically indistinguishable but genetically divergent (11). *Shigella* spp., obligatory intracellular intestinal pathogens restricted to primates, are *E. coli* closely related but distinct from phylogroups A/B1 and E (12). They have acquired a virulence plasmid (13) and undergone convergent evolution, involving both gain and loss of functions, including metabolic traits (14). The *E. coli* population structure is supported by both genetic distances (15, 16) and gene content patterns (17). Consequently, *E. coli* harbors a pan- and core-genome not only at the species level but also within phylogroups, revealing a fractal-like genetic organization. Within phylogroups, subpopulations are commonly delineated using multilocus sequence typing (MLST) (18), sometimes further grouped into biologically meaningful sequence type complexes (STcs).

Phylogroups are non-randomly associated with the different *E. coli* hosts, and host diet may shape their distribution (3), supporting the relevance of phylogroup-specific nutrient-niche adaptation. Similarly, some ST/STcs are strongly associated with specific hosts, such as STc117 (phylogroup G) with poultry (19), and Shiga toxin-producing *E. coli* (STEC) O157:H7 ST11 (phylogroup E) with livestock reservoirs (20, 21). Longitudinal studies in humans (22, 23) also revealed phylogroup-dependent colonization patterns. These findings support, in addition to niche differentiation, the existence of a trade-off between colonization and residence: phylogroups B2 and F tend to be long-term residents of the gut, while A, B1, and D display strong colonization abilities.

Another key aspect of the *E. coli* population is the strong link between phylogroups and lifestyles: commensals are mainly in phylogroups A and B1 (1), while ExPEC strains are largely found in B2, D, and F, and enterohemorrhagic *E. coli* (EHEC) strains in B1 and E (2), these associations being not mutually exclusive. This overlap, potentially resulting from epistatic interactions (2), makes it difficult to disentangle phylogroup from lifestyle-associated traits, such as metabolic pathways. Furthermore, identifying metabolic features specific to lifestyle is challenging because commensals InPEC and ExPEC all inhabit the gut and must compete for similar nutrients. These difficulties, along with the major footprint of phylogeny on metabolism, were already evident in a pioneering study of pan and core metabolism of 29 *E. coli* strains, including *Shigella* spp. strains (24). This study demonstrated that metabolic profiles were more strongly associated with phylogroups than with pathotypes, except for *Shigella* spp. strains, which displayed inactivation of metabolic networks primarily through genetic drift but also in some cases through adaptive convergent evolution (25, 26). Otherwise, only a limited set of reactions was preferentially associated with either commensals or ExPEC strains, and none were entirely specific.

Subsequent studies also aimed to identify phylogroup- or pathotype-specific metabolic profiles. For instance, using genome-scale metabolic reconstructions of 55 *E. coli* strains, Monk *et al*. identified a few pathways that could differentiate ExPEC from commensals (27). However, the presence/absence patterns of many of them were also specific to phylogroup B2, consistent with the distinct gene repertoire of this group (28). Notably, the absence of pathways such as fructoselysine and psicoselysine (Amadori products) degradation, as well as 3-(3-hydroxyphenyl)propanoate catabolism, was mainly observed in ExPEC, but it is also a hallmark of B2 strains (28, 29). However, exceptions exist. For instance, the pandemic ExPEC ST131 and the atypical B2 commensal ST452 do carry the latter pathway (22, 30), highlighting intra-phylogroup heterogeneity. Indeed, as expected due to phylogroup-specific pan- and core-genomes (17), finer-scale analyses also revealed ST-specific metabolic profiles (31, 32), adding another layer of complexity to attempts to link metabolism with lifestyle.

To date, knowledge of *E. coli* population metabolic specificities is often derived either from high-quality but limited data sets (24, 27, 33) or from larger, more comprehensive but heterogeneous collections (30). Notably, the absence of metadata regarding strain origin and context of isolation may often limit the epidemiological relevance and interpretability of findings. In this context, we analyzed a large and well-defined genome collection of *E. coli* strains, including both adult commensals and ExPEC causing bloodstream infections (BSI) from various portals of entry and ventilator-associated pneumonia, gathered during two decades (2000–2017) by our group in France. Using a pangenome approach coupled with metabolic pathway predictions, we explored metabolic features associated with both lifestyle and phylogenetic background, with particular focus on the dominant ExPEC STcs (i.e., STc131, 95, 73, 69, 10, and 14) (2, 34). Our aim was to identify metabolic traits that may be associated with strain sources and/or contribute to the epidemiological success of some STcs, under the hypothesis that metabolism is a key determinant of ExPEC major clones’ emergence and infection potential.

## MATERIALS AND METHODS

### Commensal and pathogenic strain genome data sets

We analyzed *E. coli* genomes from both commensal (*n* = 370) and extraintestinal pathogenic (*n* = 1,128) human adult strains, all isolated in France across a time period ranging from 2000 to 2017. The delineation of the lifestyle commensal versus pathogenic of the strains was based on the origin of the strain and the context of the isolation. Commensal strains came from community living volunteers with no history of gastrointestinal disease, no symptoms of immunosuppression, no antibiotic therapy in the previous month, and no hospitalization in the three months preceding inclusion (35, 36). They consisted of five collections obtained from feces or rectal swabs and gathered during prospective and multicentric studies: ROAR in 2000 (*n* = 50) (37) and LBC in 2001 (*n* = 27) (38) in Brittany; PAR in 2002 (*n* = 27) (38), Coliville in 2010 (*n* = 246) (35), and CEREMI in 2017 (*n* = 20) (39) in the Paris area. In all studies, a single *E. coli* colony per individual was randomly selected from the Drigalski agar plate used for strain isolation and retained for further analysis.

Pathogenic strains were obtained from hospitalized patients during three prospective and multicentric studies: Colibafi (*n* = 367) (40) and Septicoli (*n* = 545) (41) studies that correspond to strain isolated from BSI in the Paris area in 2005 and 2016–2017, respectively, and Colocoli, a study of *E. coli* pulmonary strains (*n* = 216) gathered in 2012–2014 from mechanically ventilated patients with pneumonia in French intensive care units (42). Blood cultures are normally sterile, and the isolation of an *E. coli* indicates an ongoing pathological process. Pneumonia strains were isolated with appropriate methods (quantitative cultures of tracheal suctioning, bronchoalveolar lavage, or protected telescoping catheter), and the diagnosis of pneumonia was made using consensus intensive care unit guidelines. A single *E. coli* colony from the culture of the clinical sample was retained per individual. Thus, among pathogenic strains, the primary source of infection was urinary (*n* = 470), digestive (*n* = 281), pulmonary (*n* = 235), or other (*n* = 142) (unknown [*n* = 57], multiple [*n* = 39], catheter [*n* = 29], skin [*n* = 9], gynecologic [*n* = 4], and surgical site [*n* = 4]).

All genomes were short-read sequenced on Illumina platforms. Phylogroups, MLST, and STcs were retrieved from the corresponding studies. Detailed information, including the corresponding bioprojects, is available in Table S1.

### Distribution of top 10 STs and phylogroups according to strain origin

To evaluate clonality across origins, we calculated the proportion of strains represented by the top 10 STs for each origin and tested the independence between these counts and sample origin using a χ^2^ test. As a post-hoc test, pairwise comparisons of these proportions were then performed with Bonferroni-adjusted *P*-values.

Associations between phylogroups and sample origin were assessed using binomial logistic regression, modeling the probability of each phylogroup by origin (digestive, pulmonary, urinary, or other), with commensal isolates as the reference. Odds ratios (ORs), 95% confidence intervals (95% CI), and *P*-values (α = 0.05) were reported. Phylogroup H and cryptic clades were excluded from these comparisons due to the very low number of strains.

### Pangenome construction

Genomes were annotated with Pyrodigal v3.2.1 (43), a Python module that provides bindings to Prodigal (44), Aragorn v1.2.41 (45), and Infernal v1.1.4 (46), and the pangenome was built using PPanGGOLiN v2.0.0 (47), applying thresholds of 80% amino acid identity and alignment coverage to define protein families. The pangenome file is available on Zenodo (48). From the multiple sequence alignment of core genes (option “MSA” of PPanGGOLiN), a phylogenetic tree was computed with iqtree v1.6.12 (49) and the GTR+F+I+G4 model, as previously described (28). Patristic distances between all genome pairs were determined from this tree with the function “cophenetic” from R package “ape” (50).

### Panreactome and pathway prediction

To determine Gene-Protein-Reaction (GPR) associations at the pangenome level, we applied a three-step approach in which representative protein sequences from each pangenome family were aligned against reference protein sequences using Diamond v2.1.8 (51) in “ultrasensitive” mode, with MetaCyc v27.0 (52) serving as the reference database for reactions and pathways. First, representative pangenome sequences were compared with *E. coli* K-12 protein sequences from EcoCyc v27.0 database (53). Only matches with at least 80% identity and coverage were considered, and EcoCyc reaction annotations with equivalents in MetaCyc were transferred to the corresponding pangenome families. Second, for representative proteins without K-12-related reactions, we compared them against MetaCyc protein sequences to transfer corresponding reaction annotation, considering only best hits with a minimum identity of 40% and coverage of 80%. Finally, for the remaining proteins, we ran kofamScan v1.3.0 (54) with the KofamKoala database (version 2023-10-02), retaining only hits above the score threshold defined for each KEGG Orthology (KO) group. To annotate pangenome families, KEGG reactions associated with each KO group were mapped to the corresponding MetaCyc reactions. If no cross-reference was found, only EC (Enzyme Commission) numbers of KO were used to assign enzymatic activities.

The resulting panreactome, comprising GPR associations described at the pangenome family level with either MetaCyc reactions or EC numbers, was used as input for Pathway Tools v27.0 (55), which was run by command line with default parameters and using PathoLogic file format for annotations. The corresponding file is available on Zenodo (56). For each predicted pathway, a completion value at the pangenome level was computed by dividing the number of predicted reactions by the total number of reactions in the pathway, excluding spontaneous reactions and also orphan ones (i.e., reactions not associated with any known gene in the MetaCyc database). Pathway completions were also computed for individual genomes from the pangenome gene family presence/absence matrix. A pathway was considered present in a given organism when its completion reached more than 50% of the maximum completion value observed at the pangenome level.

### Metabolic distances and clustering

Metabolic distances were computed as Manhattan distances between genomes based on presence/absence of the predicted pathways. A linear regression using “lm” from the R package “stats” was used to search for a correlation between metabolic and patristic distances.

We also performed a hierarchical clustering of genomes based on metabolic distances, considering only pathways with frequencies ranging from 5% to 95%, with the Ward D2 method using “hclust” from the R package “stats.”

### Multifactorial correspondence analysis

A factorial multiple correspondence analysis (MCA) was performed with FactoMineR (57) using the presence/absence of each pathway as active variables. Only pathways present in 5% to 95% of genomes were considered. Strain origins and phylogroups were used as illustrative variables. The results were plotted using Factoextra (58), considering the first two eigenvalues. The same analysis was run using the presence/absence of reactions as active variables.

### Genomic characterization of D-apiose degradation gene cluster

To characterize D-apiose degradation gene cluster, we used the MicroScope platform (59) and Clinker (60) to compare gene clusters from *Pectobacterium carotovorum* WPP14 (Refseq accession number: GCF_013488025.1), where the pathway has been previously described (61), *Escherichia fergusonii* ATCC35469 (Refseq accession number : GCF_000026225.1), *E. coli* H1-004-0008-M-Y (ST131 025b-H4-fimH30 – Clade C1), *E. coli* H1-003-0083-B-J (ST131 025b-H4-fimH30 – clade C2), and *E. coli* H1-002-0016-H-R [ST1193 (STc14)]. This approach enables the comparison of each coding sequence at the protein level and the analysis of synteny conservation.

In a second step, we analyzed the location of the gene cluster among all *E. coli* complete genomes available in RefSeq on September 19, 2022 (*n* = 2,302) (62), as well as other *Escherichia* non-coli species (*n* = 167). Nucleic sequences spanning from *entH* to *cusS* were extracted and annotated using Prokka v1.13.3 (63). From the pangenome computed with PPanGGOLiN, a subgraph of this genomic region was extracted and visualized with Gephi (64). The pangenome file is available on Zenodo (65). Using this complete genome data set, we also conducted several phylogenetic analyses with iqtree v1.6.12, including (i) a core-gene phylogeny at the *Escherichia* genus level, (ii) core gene-based phylogenies restricted to *E. fergusonii* and to *E. coli* phylogroup B2, (iii) a phylogeny of the D-apiose degradation gene cluster, and (iv) three additional trees focusing on genes neighboring the apiose gene cluster: *fepA*, *entD,* and *ybdG*. Core-gene phylogenies were inferred using the GTR+F+I+G4 model, while the most appropriate model for all other alignments was selected with ModelFinder (66). Patristic distances were computed from the trees using the “cophenetic” function from the R package “ape.”

### Large-scale screening of D-apiose degradation gene cluster

We performed a large-scale screening for the presence of the D-apiose degradation gene cluster among genome assemblies from the AllThebacteria database (67). In a first step, we performed a protein blast search using diamond v2.1.8 (minimum identity: 40%; minimum coverage: 80%). Protein sequences from *E. coli* SE15 (B2, ST131) (RefSeq accession number: NC_013654.1; gene locus tag ECSF_RS02670 to ECSF_RS02715) were queried against the 2,438,285 genomes with available annotations. The D-apiose degradation pathway was considered present when at least 5 of the 10 genes were detected, including the kinase *aplK* (ECSF_RS02700) and the two transketolases *aptA* (ECSF_RS02710) and *aptB* (ECSF_RS02715), which had to be located within a window of 10 consecutive coding sequences. For all genomes assigned to *E. coli* in AllTheBacteria, sequence types (STs) were determined using mlst v2.23.0 and the Warwick scheme (18, 68, 69).

In a second step, we built an *ad-hoc* database for Abricate v1.0.1 (70) using nucleic sequences of the D-apiose degradation cluster from *E. coli* SE15. We then performed a nucleotide blast search (minimal nucleotide identity and coverage: 50%) to detect sequences closely related to those found in the genus *Escherichia*.

### Growth under D-apiose-containing M9 minimal media

*P. carotovorum* WPP14, *E. fergusonii* ATCC 35469, three *E. coli* strains carrying the apiose gene cluster (H1-004-0008-M-Y, H1-003-0083-B-J, and H1-002-0016-H-R), and *E. coli* K-12 MG1655 were grown aerobically in LB medium at 37°C overnight. Bacterial cells were then washed and diluted 1:1,000 into M9 minimal medium supplemented with 10 mM apiose (Omicron) as the sole carbon source. Growth was monitored over 48 h at 37 °C under continuous shaking by automatic measurement of optical density (OD) at 600 nm.

## RESULTS

### An epidemiologically relevant collection with lifestyle-specific population genetic structure

The *E. coli* collection used in this study comprises 1,498 genomes. Overall, it covers the eight main *E. coli* phylogroups, along with one strain from phylogroup H (2) and eight from *Escherichia* cryptic clades (Table S2). Although cryptic clade I (*n* = 3) belongs to *E. coli*, the other clades correspond to the following two new species of the genus *Escherichia: E. ruysiae* (clades III and IV) (*n* = 2) and *E. marmotae* (clade V) (*n* = 3) (11, 14, 71, 72).

**Fig 1:**
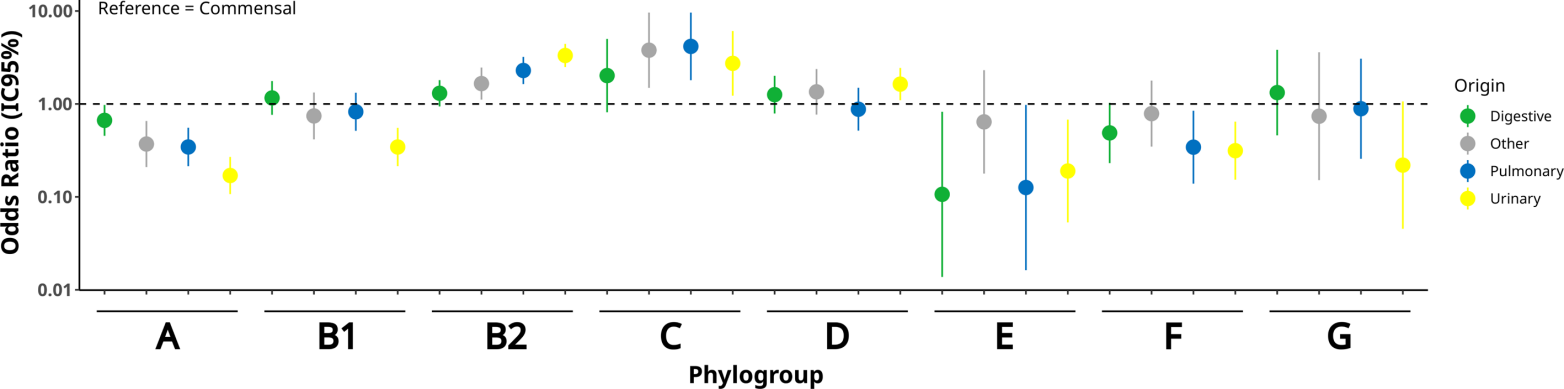
Distribution of phylogroups according to strain origin. Odds ratios (ORs) for the association between *E. coli* phylogroups and sample origins were estimated using binomial logistic regression models, with the commensal origin as the reference category. ORs are shown as points, with the 95% confidence intervals represented by vertical bars. Colors indicate the strain origins. The dotted line corresponds to an OR of 1.0, indicating no difference relative to the commensal origin.

We classified the extraintestinal pathogenic strains according to their clinical origin: BSI with urinary (*n* = 470) or digestive (*n* = 281) portal of entry, the two main portals of entry of BSI, BSI with diverse portal of entry (*n* = 142), and pneumonia-associated BSI and pneumonia in patients receiving mechanical ventilation (*n* = 235). Several previously reported associations between phylogroups and isolation sources were observed (1, 73–76). Compared with commensal strains, several phylogroups were underrepresented: phylogroup A across all origins, phylogroup E in all origins except BSI of “other” origin, phylogroup F in urinary and pulmonary origins, and phylogroup B1 in urinary origin (Fig. 1, Table S2). Conversely, phylogroup B2 was overrepresented in all origins except digestive, and phylogroup D was overrepresented in urinary origin. The proportion of strains belonging to the top 10 STs also varied significantly by origin, indicating higher diversity in commensal strains compared with pulmonary (*P* < 0.001) and urinary strains (*P* < 0.001) (Table 1, Tables S3 and S4). Strains from digestive (*P* < 0.001) and other origins (*P* = 0.015) likewise showed greater diversity than the urinary origin.

**TABLE 1:** Proportion of strains belonging to the top 10 STs according to their origin^*a*^

Origin	Commensal N (%)	Urinary N (%)	Digestive N (%)	Pulmonary N (%)	Other N (%)
Top10 STs	155 (41.89)	325 (69.15)	138 (49.11)	138 (58.72)	77 (54.23)
Other STs	215 (58.11)	145 (30.85)	143 (50.89)	97 (41.28)	65 (45.77)

^*a*^ The detailed composition of the top 10 STs is provided in Table S3, and the results of the pairwise comparisons in Table S4.

Thus, our data set is phylogenetically diverse, epidemiologically relevant, and exhibits a lifestyle-associated population structure, making it meaningful to search for metabolic associations.

### A collection with a high genomic and metabolic diversity

**Fig 2:**
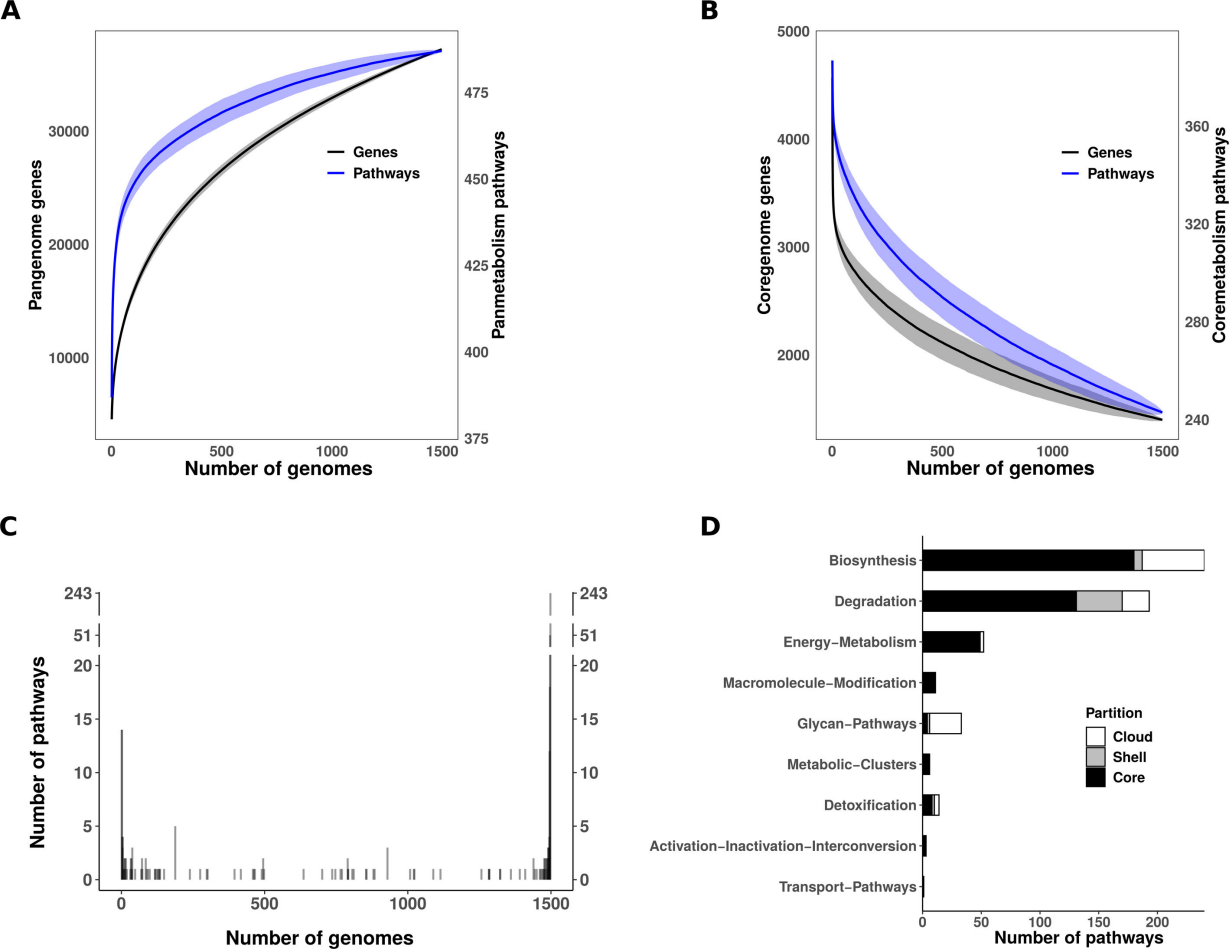
*E. coli* core- and pan-metabolism. Evolution of (**A**) the pangenome and panmetabolic pathways, and (**B**) the core genome and core metabolic pathways as a function of the number of included genomes. To account for genome variability, 1,000 random permutations were performed at each step of genome addition. The resulting mean number of genes and pathways is shown in black and blue, respectively. Shaded areas represent the standard deviation. (**C**) Frequency of pathways across the 1,498 genomes analyzed. Pathways on the left side of the graph are present in only one genome (*N* = 14, 2.87% of panmetabolic pathways), while those on the right side are found in all genomes (*n* = 243, 49.90% of panmetabolic pathways). For the sake of readability, the y-axis is broken. (**D**) Distribution of pathways among the main metabolic functional categories defined by MetaCyc. Bar plots are colored according to pathway frequency. Core (black), shell (gray), and cloud (white) partitions correspond to pathways with frequencies f ≥ 95%, 15% ≥ f > 95%, and f <15%, respectively.

We then analyzed the diversity of the whole collection in terms of gene families, reactions, and metabolic pathways (Fig. 2). The rarefaction curves revealed relatively closed panreactome (Fig. S1A) and panmetabolic pathway repertoires (Fig. 2A), comprising 3,023 reactions and 487 predicted pathways, in contrast to a much larger and more open pangenome consisting of 37,236 gene families. Reactions and pathways appeared more conserved than genes, reaching core values of 1,360 reactions (44.99%) and 243 pathways (44.90%), respectively, compared with 1,400 core genes (3.76%) (Fig. 2B; Fig. S1B). The frequency distribution of reactions and pathways displayed a U-shape (Fig. 2C; Fig. S1C), inverted compared to the typical gene frequency distribution (77), with a higher proportion of conserved elements compared to unique or rare ones. A large number of variable pathways were associated with the “Biosynthesis” and “Glycan Pathways” categories, with an overrepresentation of pathways involved in O-antigen biosynthesis, present in 27/60 (45.0%) and 27/29 (93.1%) of the variable pathways in these categories, respectively (Fig. 2D). Another major subset of the variable pathways (62/161, 38.5%) was linked to “Degradation” processes.

### Metabolic diversity is mainly driven by phylogeny

**Fig 3:**
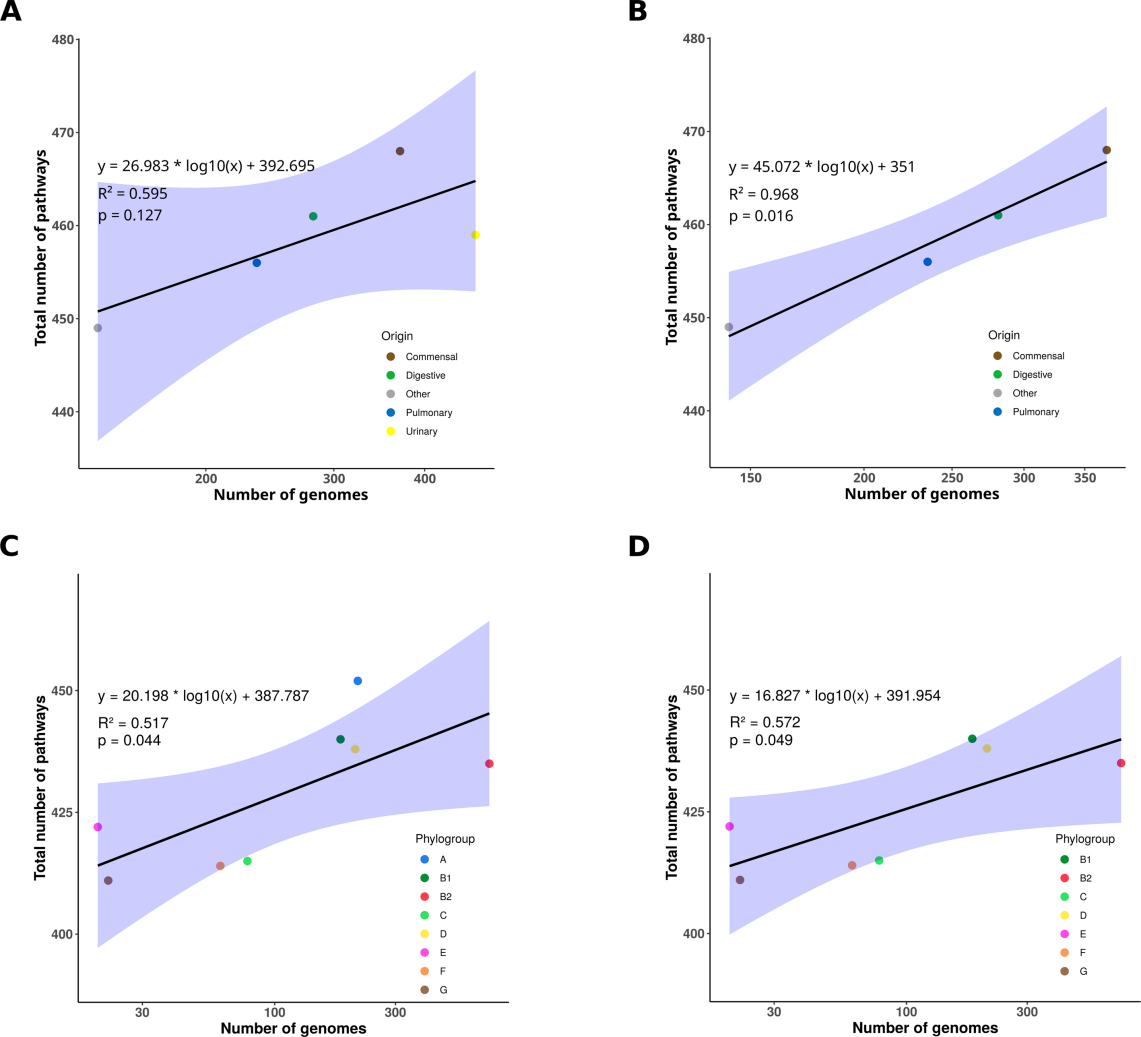
Correlation between the number of pathways and genomes. Total number of pathways as a function of the number of genomes: (**A**) from all sources, (**B**) excluding those of urinary origin, (**C**) from all phylogroups, and (**D**) excluding phylogroup A. Data points are colored according to their origin or phylogroup. The regression line is shown in black, with the 95% confidence interval in blue. Each graph includes the linear regression equation, the coefficient of determination (R^2^), and the *P*-value.

In the second step, we compared the sizes of the pangenome, panreactome, and panmetabolic pathways relative to the number of strains, taking into account their origin and phylogroup. Overall, when all sources were included, no significant correlation was observed between the number of genomes and the number of genes, reactions, or pathways (Fig. 3A; Fig. S2A and S2B; Table S5). However, excluding strains of urinary origin markedly improved the correlation, which became significant for both reactions and pathways (Fig. 3B; Fig. S2C and S2D). When analyzed by phylogroups, significant correlations were observed for gene, reaction, and pathway counts (Fig. 3C; Fig. S2E and S2F; Table S5). Notably, excluding phylogroup A strains further strengthened these correlations in most cases (Fig. 3D; Fig. S2G and S2H). Together, these results suggest a reduced metabolic and genomic diversity among strains isolated from urinary-source BSI, while strains from phylogroup A appear to harbor increased diversity.

**Fig 4:**
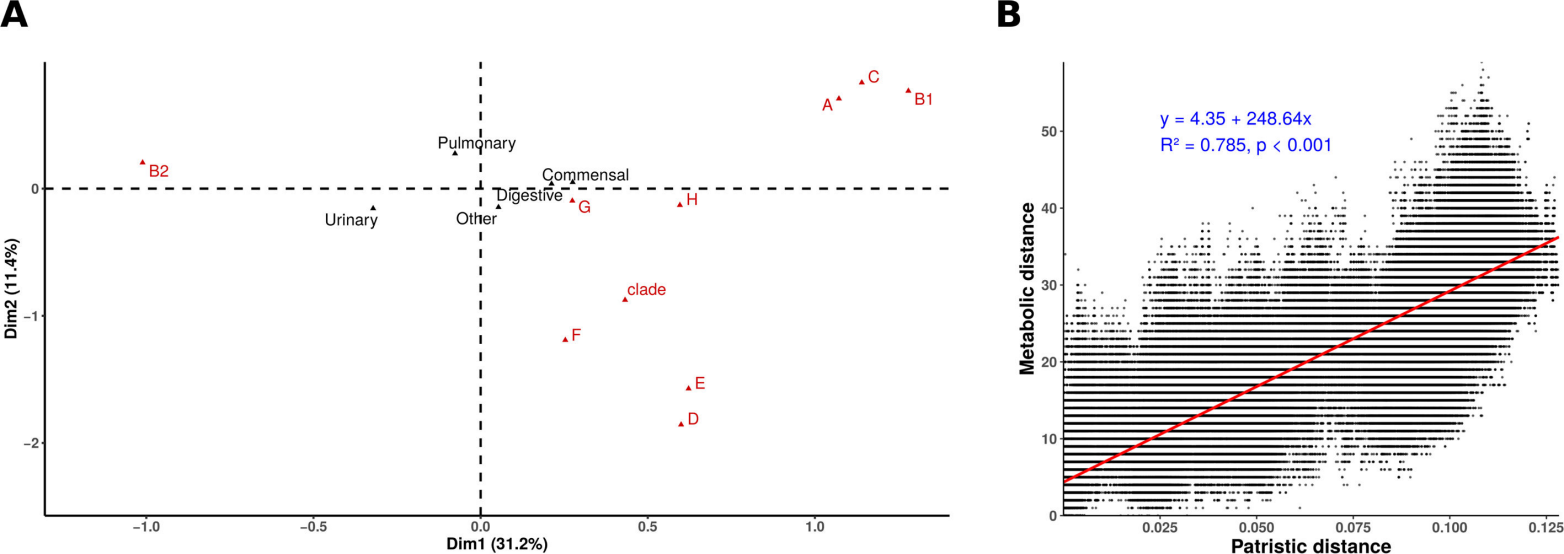
Correlation between phylogeny, origin, and metabolism. (**A**) Multiple correspondence analysis of pathway occurrences among the 1,498 genomes analyzed. The x- and y-axes represent the first two dimensions, which together account for 42.6% of the variability. Phylogroups (in red) and origin (in black) are shown as illustrative variables. (**B**) Correlation between patristic and metabolic distances. Patristic distances represent the branch lengths between genome pairs in the core gene-based phylogenetic tree. Metabolic distances are Manhattan distances computed from the binary pathway presence/absence matrix. Each point represents a pair of genomes. The regression line is shown in red, while the linear regression equation, coefficient of determination (R^2^), and *P*-value are shown in blue. Only strains belonging to *E. coli sensu stricto* are included in panel B (i.e., *Escherichia* clade strains are excluded).

We then investigated whether specific pathway and reaction presence/absence profiles could segregate strains based on their phylogroup and/or isolation source. To this end, we performed a multifactorial correspondence analysis (MCA) using the presence/absence matrix of metabolic pathways (Fig. 4A) or reactions (Fig. S3) across the 1,498 strains. The first two dimensions of the MCA captured a substantial proportion of the overall variability, accounting for 42.6% for pathways and 41.8% for reactions. When illustrative variables were mapped onto the MCA plots, metabolic diversity appeared primarily structured by phylogeny rather than origin. Specifically, the first dimension mainly separated phylogroup B2 from phylogroups A, B1, and C, while the second dimension distinguished phylogroups D, E, and F from the others. No clear separation was observed between strains based on their origins along these axes. As recently shown (78), digestive-origin BSI strains can be subdivided into biliary and abdominal groups, resembling commensal and UTI strains, respectively. We applied this classification to our data set and mapped it onto our MCA. As expected, the biliary group clustered closer to phylogroups A, B1, and C, whereas the abdominal group was positioned nearer to urinary-origin strains (Fig. S4).

These findings are also consistent with the observed correlation between metabolic and patristic distances computed across all genome pairs (Fig. 4B), excluding cryptic clades. The inclusion of *Escherichia* cryptic clades disrupted the correlation, likely due to their substantial genetic divergence from *E. coli sensu stricto* (Fig. S5).

### A limited number of pathways separate phylogroups and major STcs responsible for BSI

**Fig 5:**
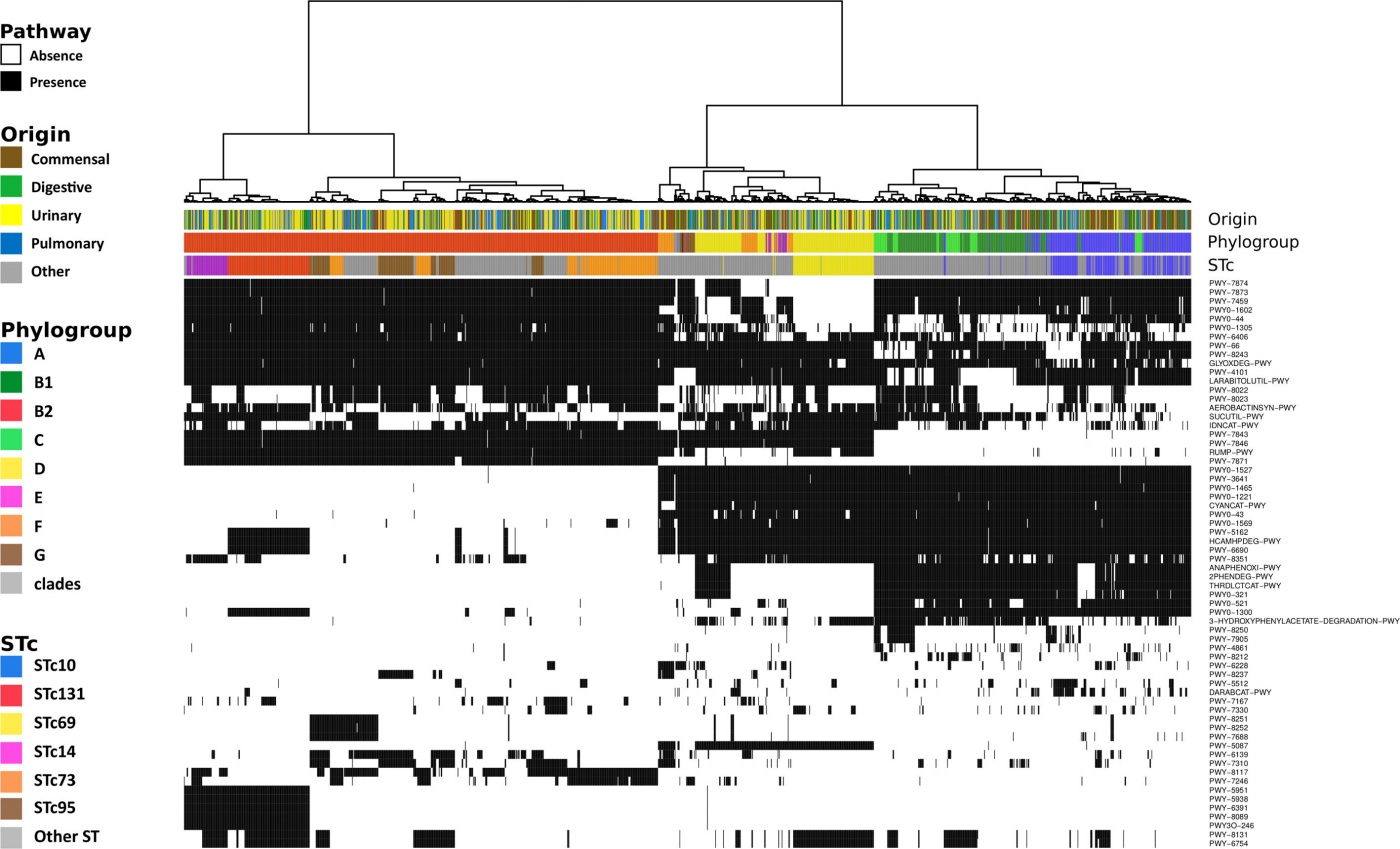
Hierarchical clustering of metabolic pathways and associations with origins, phylogroups, and major ExPEC STCs. The dendrogram above the heatmap represents the clustering of genomes according to their pathway content. Clustering was performed with the Ward D2 method on Manhattan distances based on the presence and absence of metabolic pathways having a frequency between 5% and 95%, shown in black and white on the heatmap, respectively. From top to bottom, the color strips indicate the origin, phylogroup, and STc of each strain. Only the most prevalent STcs associated with bacteremia (79), along with the emerging high-risk clone STc14 (34), are highlighted in color; all other STs are shown in gray.

The lack of a global correlation between isolation source and pathway presence/absence does not rule out the possibility that a small subset of metabolic pathways play specific roles in adaptation or pathogenesis. Indeed, analyzing all pathways collectively may have masked more subtle associations. To explore this, we focused on metabolic pathways with intermediate frequencies (5%–95%), excluding both highly conserved and very rare ones. Analysis of the presence/absence profiles of these 64 pathways did not reveal any segregation based on strain origin (Fig. 5). Instead, the patterns strongly mirrored phylogroup classification. Phylogroup B2 appeared particularly distinct from others, largely due to the absence of multiple pathways.

At a finer resolution, specific metabolic signatures were observed in major STcs associated with BSI. Notably, STc131 and STc14 (both from phylogroup B2), as well as STc69 (from phylogroup D), formed well-defined clusters on the heatmap, suggesting conserved and distinctive metabolic profiles within these clinically significant lineages.

To identify a limited set, or ideally unique, metabolic pathways strongly associated with pathogenicity, BSI portal of entry, or phylogroup, we next examined the frequency of individual metabolic pathways across these variables. We specifically searched for pathways that were overrepresented (>80% of frequency) in at least one group and underrepresented (<20% of frequency) in at least another, across the following comparisons: (i) commensal versus pathogenic strains, (ii) strains stratified by BSI portal of entry, and (iii) phylogroups. No pathway fulfilled these criteria when comparing lifestyles (commensal, pathogen, or BSI portal of entry). However, 34 pathways were found to be either overrepresented or underrepresented in at least one phylogroup (Table S6). All but one of these pathways was associated with degradation processes. Interestingly, some of these pathways, such as heme degradation IV and V (PWY-7843 and PWY-7846), encoded by the *chu* gene cluster, are the basis of Clermont’s phylogrouping method (80, 81) and separate A, B1, and C (absence) from others (presence).

We used the same approach to investigate whether specific pathways were enriched within particular STcs relative to the rest of their respective phylogroup. No such pathways were found for STc10 compared to other phylogroup A strains, in line with the extensive genetic and metabolic diversity of this group. In contrast, three pathways were found to be specifically enriched in STc69 (phylogroup D) compared to other strains within the same phylogroup: S-methyl-5'-thioadenosine degradation I, 5′-deoxyadenosine degradation II, and formaldehyde oxidation I (Table S7). The first two have been recently described in a dihydroxyacetone phosphate shunt, enabling the recycling of 5′-deoxynucleosides, which may accumulate in urine and blood in humans (82, 83).

Also, among the major STcs responsible for extraintestinal diseases within phylogroup B2, namely STc131, STc73, STc95, and STc14, we identified 13 pathways with distinct presence/absence patterns (Table 2). Most of these were involved in degradation (8/13), followed by biosynthesis processes (3/13). Three of the STc131-specific pathways were linked to the *mhp* gene cluster (HCAMHPDEG-PWY, PWY-5162, and PWY-6690) (22). STc95 was characterized by the presence of the CMP-N-acetylneuraminate biosynthesis II pathway, consistent with the known high frequency of type II capsule production in this lineage (84). Other STc-specific pathways included 2-O-α-mannosyl-D-glycerate degradation (STc131), pectin degradation II (STc73), D-glucosaminate degradation (STc95), and sulfoquinovosyl diacylglycerides and sulfoquinovosyl glycerol degradation (STc14). Additionally, five pathways were shared between STc131 and STc14, with identical frequencies. Closer inspection revealed that these were all predicted based on reactions involved in D-apiose degradation, suggesting a shared acquisition of this specific catabolic capacity.

**TABLE 2:** Pathways specifically absent/present among the major ExPEC STc from phylogroup B2^*a*^

Pathway ID	Pathway name	Metacyc classification^*b*^	Pattern^*c*^	STc73 (*N* = 170)	STc95 (*N* = 132)	STc131 (*N* = 122)	STc14 (*N* = 59)	Other ST phylogroup B2 (*N* = 222)	Notes
HCAMHPDEG-PWY	3-phenylpropanoate and 3-(3-hydroxyphenyl)propanoate degradation to 2-oxopent-4-enoate	Degradation	Presence STc131	0	0	100	0	8.11	Part of the *mhp* gene cluster
PWY-5162	2-oxopentenoate degradation	Degradation	Presence STc131	0	0	100	0	8.11	Part of the *mhp* gene cluster
PWY-6690	cinnamate and 3-hydroxycinnamate degradation to 2-hydroxypentadienoate	Degradation	Presence STc131	0	0	100	0	8.11	Part of the *mhp* gene cluster
PWY-6139	CMP-N-acetylneuraminate biosynthesis II	Biosynthesis	Presence STc95	0	99.24	1.64	8.47	39.64	Group two capsule synthesis
PWY3O-246	(R,R)-butanediol degradation	Degradation	Presence STc131, STc14	0	0	100	100	2.7	Part of D-apiose degradation
PWY-5938	Pyruvate fermentation to (R)-acetoin I	Energy-Metabolism	Presence STc131, STc14	0	0	100	100	2.7	Part of D-apiose degradation
PWY-5951	(R,R)-butanediol biosynthesis	Biosynthesis	Presence STc131, STc14	0	0	100	100	2.7	Part of D-apiose degradation
PWY-6391	Meso-butanediol biosynthesis I	Biosynthesis	Presence STc131, STc14	0	0	100	100	2.7	Part of D-apiose degradation
PWY-8089	D-apiose degradation I	Degradation	Presence STc131, STc14	0	0	100	100	2.7	Part of D-apiose degradation
PWY0-1300	2-O-α-mannosyl-D-glycerate degradation	Degradation	Presence STc131	0	0	100	0	10.36	
PWY-7246	Pectin degradation II	Glycan-Pathways	Presence STc73	97.06	0	0	23.73	10.36	
PWY-7310	D-glucosaminate degradation	Degradation	Presence STc95	0	100	0	0	26.13	
PWY-8351	Sulfoquinovosyl diacylglycerides and sulfoquinovosyl glycerol degradation	Degradation	Presence STc14	0	0	21.31	100	32.43	

^*a*^ Frequencies (%) of pathways are presented for each major ExPEC STc and other ST from phylogroup B2.

^*b*^ Level 1 from Metacyc classification.

^*c*^ Pattern defined as minor presence/absence of the pathway among the 4 ExPEC STc from phylogroup B2.

In sum, only a few metabolic pathways appear to be specifically associated with the major STcs responsible for bacteremia. Strikingly, in phylogroup B2, most of these pathways are involved in the degradation of plant-derived compounds, pointing to a possible ecological adaptation of these successful clones through the acquisition of specialized catabolic functions. Two other notable pathways identified in STc69 may contribute to growth outside the intestinal niche, particularly during the infection process, for instance, in urine.

### Characterization of the D-apiose degradation pathway in *Escherichia* spp

**Fig 6:**
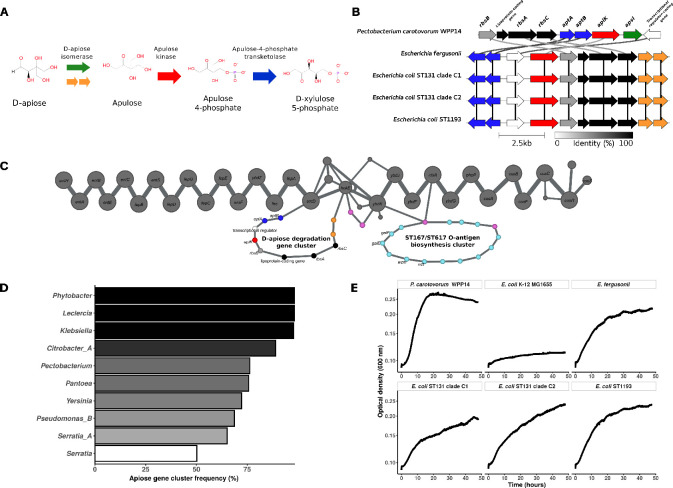
Genomic and phenotypic characterization of the D-apiose degradation pathway. (**A**) Schematic representation of the D-apiose degradation pathway (MetaCyc ID: PWY-8089). Reactions are indicated by colored arrows, adapted from the color scheme proposed by Carter *et al*. (61). The two putative oxidoreductases, hypothesized to functionally replace the D-apiose isomerase in *Escherichia* genus, are highlighted in orange. (**B**) Comparison of the apiose gene cluster in *Pectobacterium carotovorum* WPP14 (Assembly accession: GCF_013488025.1), *E. fergusonii* ATCC 35469 (Assembly accession: GCF_000026225.1), *E. coli* H1-004-0008-M-Y (ST131 O25b:H4-fimH30 – clade C1), *E. coli* H1-003-0083-B-J (ST131 O25b:H4-fimH30 – clade C2), and *E. coli* H1-002-0016-H-R (ST1193). Genes are depicted as arrows, with colors corresponding to the reactions in panel A. Shading between genes in neighboring clusters indicates protein sequence identity (0% = white; 100% = black). The figure was generated using Clinker (60). (**C**) Pangenome graph representation of the genomic region spanning *entH* to *cusS* in complete genomes of *E. coli* (*n* = 2,302) and other *Escherichia* species (*n* = 167). Nodes represent gene families, and edges represent their genomic neighborhood. Node and edge sizes are proportional to their frequency in the data set. Nodes corresponding to the apiose gene cluster are colored as in panel B; transposase-related nodes are shown in pink, and those corresponding to the ST167/ST617 O-antigen biosynthesis cluster are shown in light blue. For the sake of readability, only edges found in more than 2% of genomes are represented. (**D**) Large-scale genome screening for the presence of apiose cluster-related genes. Bar plots show the frequency of apiose degradation-associated genes in the AllTheBacteria database. Only genera represented by more than 50 genomes and with a gene cluster frequency of at least 50% are shown. The taxonomy, retrieved from AllTheBacteria database, is determined based on GTDB (85). (**E**) Growth curves using D-apiose as the sole carbon source. Growth was monitored by automated OD600 measurements over 48 h at 37°C. *P. carotovorum* WPP14 and *E. coli* K-12 MG1655 were used as positive and negative controls, respectively. All other tested strains, carrying the apiose gene cluster, correspond to those shown in panel **B**.

We then focused on the *D*-apiose degradation pathway (Fig. 6A), as it emerged as a core metabolic feature uniquely shared between the two most recent pandemic *E. coli* clones, STc131 and STc14, and absent in the rest of *E. coli* species. Screening complete genomes from the RefSeq database confirmed its presence in 100% of STc131 (*n* = 126) and STc14 (*n* = 22) strains, as well as in 19 of 74 (25.68%) *E. fergusonii* genomes. A few other sequence types also carried the apiose pathway gene cluster, including ST583 (*n* = 2), ST788 (*n* = 2), ST636 (*n* = 1), ST7898 (*n* = 1), and ST122 (*n* = 1) (Fig. S6). All of them belong to phylogroup B2.

Gene cluster comparison among representative strains of *E. fergusonii* (ATCC 35469), *E. coli* STc131 clade C1 (H1-004-0008-M-Y), STc131 clade C2 (H1-003-0083-B-J), and STc14 (H1-002-0016-H-R, ST1193) revealed an identical gene organization (Fig. 6B). While similar to the cluster described in *Pectobacterium carotovorum* WPP14 (61), the *Escherichia* version did not encode the *apsI* gene coding for a D-apiose isomerase. Instead, it featured two coding sequences (CDSs) encoding putative oxidoreductases, which we hypothesize functionally replace *apsI* in this genus.

To further investigate the genomic context of the apiose cluster, we constructed a pangenome from all complete *Escherichia* genomes (Table S8). In all cases, the cluster was located downstream of the *enterobactin* biosynthesis genes (Fig. 6C). Both upstream and downstream regions were highly conserved across the *Escherichia* genus. Interestingly, a distinct locus encoding an O*-*antigen biosynthesis cluster, previously described as specific to ST167-ST617 from phylogroup A (86), was also found in this genomic region. Patristic distances computed from the apiose gene cluster phylogeny showed strong congruence with those from the core-gene phylogeny of *E. coli* phylogroup B2 (Fig. S7A). This finding is consistent with the exact correspondence between the apiose cluster phylogeny and the structure of the main ST131 clades A, B, and C1/C2 (Fig. S8). In contrast, no such congruence was observed for *E. fergusonii* (Fig. S7B). We next examined the phylogenies of core genes flanking the D-apiose cluster. The *ybdG*-based tree showed strong congruence with the *Escherichia* species phylogeny (Fig. S9). Conversely, the *entD* phylogeny revealed a split within the *E. fergusonii* population, with apiose-positive genomes clustering among the B2 apiose-positive clones (Fig. S10). Finally, in the *fepA* phylogeny, all *E. fergusonii* genomes formed a single group that clustered together with clade I, positioned between *E. coli* phylogroups D/F/G and B2 (Fig. S11).

We then wanted to evaluate the broader taxonomic distribution of the D-apiose degradation gene cluster, through screening of the AllTheBacteria database. The pathway was predicted in 137,045/2,438,285 genomes (5.62%), spanning 98 genera and 364 species, most of which are environmental or plant-associated bacteria such as *Leclercia*, *Phytobacter*, *Klebsiella*, *Citrobacter*, and *Pectobacterium* (Fig. 6D). Within the *Escherichia* genus, the pathway was detected in 31,863/399,870 *E. coli* genomes (7.97%), 6/29 *E. fergusonii* (20.69%), and none of *E. albertii*, *E. marmotae*, *E. ruysiae*, *E. whittami*, or *E. sp005843885*. It was also present in 6/7 genomes (85.71%) of *E. sp002965065*, a yet unnamed species phylogenetically related to other cryptic clades. Within *E. coli*, the pathway was almost always present in ST131 (24,247/24,580; 98.65%) and in the two main STs from STc14, ST14 (363/364; 99.73%), and ST1193 (3,487/3,521; 98.78%). Then, to investigate a potential progenitor, we compared apiose-related genes from *Escherichia* and non-*Escherichia* genera. However, apart from a few low-quality genomes showing signs of contamination, no non-*Escherichia* genome showed matches above 90% nucleotide identity to the *E. coli* apiose genes, and conversely, no *Escherichia* genome showed matches below 90% identity to these genes (Fig. S12).

Finally, to experimentally validate the predicted phenotype, we performed growth assays in M9 minimal medium supplemented with D-apiose as the sole carbon source. While *E. coli* K-12 failed to grow, the positive control (*P. carotovorum*) and all apiose cluster-positive strains, including *E. fergusonii* (ATCC 35469), STc131 clade C1 and C2, and STc14, successfully grew under these conditions, consistent with a functional D-apiose utilization (Fig. 6E).

Together, these data demonstrate the specific and functional presence of the D-apiose degradation pathway in STc131 and STc14. The strong phylogenetic congruence observed within phylogroup B2 and across the major STc131 clades associated with the basal position of STc131/STc14 suggests an ancient acquisition in these lineages at the emergence of the B2 phylogroup, followed by several losses in other B2 strains. In addition, the intermediate frequency of the pathway in *E. fergusonii*, along with the phylogenies of the neighboring genes, suggest an horizontal gene transfer event by interspecies recombination from *E. coli* to *E. fergusonii,* implicating the apiose gene cluster and flanking core genes and its propagation within *E. fergusonii* by homologous recombination as previously reported for the high pathogenicity island (87).

## DISCUSSION

*E. coli* is the main causative agent of both community- and hospital-acquired BSI (88), associated with a substantial burden (89). Despite advances in care, mortality remains high, ranging from 10% to 30% (41, 84, 89–93). The two main portals of entry are the urinary tract and the digestive tract, the first one being the most frequent but the least severe (40, 90). Pneumonia is the third BSI portal of entry in terms of frequency (84). Ventilator-associated pneumonia due to *E. coli* represents an underestimated disease with a 10% mortality (42). Each portal of entry of infection corresponds to a specific pathophysiology process in which *E. coli* has to face specific environments. However, *E. coli* is also a gut commensal in humans and other vertebrates (1, 3), highlighting its high intrinsic capacity for colonization. Indeed, colonization of the digestive tract is a prerequisite for both commensal and pathogenic strains. While the so-called virulence factors have been shown to increase the fitness of ExPEC in the gut (94), differences in metabolic capacities may also play a pivotal role in their successful intestinal colonization. Recent longitudinal studies have shown that *E. coli* lineages adopt diverse colonization strategies, with both residence-colonization trade-off and niche differentiation processes (22, 23). A better understanding of these adaptive mechanisms, at both genomic and metabolic levels, is essential to disentangle the respective contributions of lifestyle (commensal versus pathogenic), BSI source, and phylogenetic background.

To address this, we analyzed the metabolic diversity of *E. coli* using a well-characterized data set of commensal and ExPEC strains responsible for severe diseases, collected from prospective studies over matched time periods. Considering the B2 phylogroup strain prevalence as a proxy of the population structure, the presented data set showed proportions of B2 phylogroup strains of 32.4% in commensal isolates and 51.9% in BSI- and pneumonia-associated isolates, consistent with the epidemiology reported in other high-income countries (Europe, North America, and Australia) (1, 2, 95–100).

The central finding of this work is that no specific metabolic pathway was uniquely associated with commensal, ExPEC lifestyle, or BSI portal of entry. Our prediction approach leveraged the comprehensive annotation of *E. coli* K-12 pathways, while also extending our capacity to identify poorly characterized or novel pathways through more generalist databases. We confirmed a substantial metabolic diversity across the species, a recently recognized hallmark of *E. coli* among *Enterobacteriaceae* (101), although it remains markedly lower than the genomic diversity. We report a pan- and core-metabolism larger and smaller than in previously smaller-scale studies (24, 27), with 487 and 243 pathways identified, respectively. The distribution of pathway frequencies followed an asymmetric U-shaped curve, opposite to that of gene presence/absence patterns (17, 77), consistent with a faster signal saturation for pathways than for genes. Although this pattern may arise from unidentified pathways, the relatively restricted ecological niche of *E. coli* may involve a limited set of highly specific metabolic functions. Variable pathways were mainly involved in biosynthesis and degradation, in line with previous reports (24, 27, 101), and likely play roles in environmental adaptation and substrate use as recently shown for rare metabolic genes (102). Another significant portion of the variable metabolism was associated with O-antigen biosynthesis (27). Of note, the *rfb* gene cluster, involved in the biosynthesis of O-antigen, is a hotspot of recombination in *E. coli* (77), and we previously found major variability in these antigens among the main BSI clones (79), possibly reflecting immune-driven selective pressures.

We then examined metabolic differences associated with strain origin or phylogeny. Consistent with pangenome analysis showing that generalist phylogroups A and B1 harbor larger pangenomes despite smaller genome sizes (28), our results show increased pathway and reaction numbers in phylogroup A. Conversely, strains from urinary tract-related BSI had fewer reactions and pathways. However, MCA and heatmap analyses revealed only weak clustering by origin, underscoring the dominant influence of phylogeny. Indeed, more than 61% of urosepsis strains belonged to phylogroup B2, and nearly 70% to just 10 STs.

Overall, we observed multiple pathways enriched or depleted in specific phylogroups, nearly all related to degradation, hinting at a role in niche specialization (4, 23, 101). Pathway distribution aligned with phylogroup divisions reported in pangenome analyzes (17, 103), suggesting that metabolism in *E. coli* has followed the species evolutionary history. In agreement with earlier studies (24, 29, 30, 77), phylogroup B2 exhibited distinct metabolic features, including frequent absence of degradation pathways for 3-(3-hydroxyphenyl)propanoate, fructoselysine, psicoselysine, and putrescine II.

Due to its commensal and/or opportunistic pathogen lifestyle, *E. coli* must adapt to its primary habitat (colonization) and to extraintestinal sites (infection). Although it has been suggested that the same factors may contribute to both capacities (94), we identified ecological relevance of clone-specific pathways in both types of adaptation among B2 and D major ExPEC STcs. In STc69, three specific pathways were detected, including S-methyl-5'-thioadenosine degradation I and 5'-deoxyadenosine degradation II, recently identified in ExPEC (82, 83). These pathways enable carbon and sulfur salvage from MTA and 5’-deoxyadenosine via a dihydroxyacetone phosphate shunt. Since these substrates are present in urine and blood, they may support ExPEC growth in extraintestinal environments. Although not significantly enriched in ExPEC overall, these pathways were highly prevalent in the pandemic clone STc131 (81.97%) (Table S9).

Alternatively, among the 13 B2-specific STc pathways, 10 were involved in degradation of plant-derived products: 3-(3-hydroxyphenyl)propionate degradation in STc131 (22, 28, 30, 104); pectin degradation in STc73, a major STc usually devoid of antibiotic resistance (2); sulfoquinovosyl diacylglycerides and sulfoquinovosyl glycerol degradation in STc14, a pandemic clone whose recent emergence mimics STc131 in several respects (34); and D-apiose degradation in both STc131 and STc14, which are two of the most recently emerged pandemic clones (34, 97). These findings echo Freter’s hypothesis and recent reports of lineage-specific metabolism in the *Klebsiella pneumoniae* species complex (105) and suggest that such pathways may offer ecological advantages to each of these clones.

Among these pathways, the D-apiose degradation is of particular interest. While its presence in *E. coli* was previously proposed by Carter *et al*. based on sequence similarity (61), its highly specific distribution in STc131 and STc14 had not been reported. The conserved genomic location in both STcs, together with the congruence between the apiose gene cluster and the B2 core-gene phylogenies, supports an ancient origin within *E. coli* at the emergence of B2 strains. Analysis of the neighboring gene *entD*, a core gene involved in enterobactin biosynthesis (106), suggests that it forms part of the acquired region that may have spread through horizontal transfer by interspecies recombination, particularly among some *E. fergusonii* strains. The absence of closely related sequences in non-*Escherichia* genera further argues that the presence of the pathway in *E. coli* did not result from a recent event, although this remains subject to potential biases in the database used. Additionally, our broader screening confirmed the presence of the pathway in several genera and its high prevalence among phytopathogenic or plant-associated bacteria, consistent with the origin of apiose, a branched-chain pentose and a key component of primary cell walls in higher plants (107). Interestingly, several human gut anaerobes, including *Bacteroides* and *Clostridia*, have also been shown to degrade apiose (61), suggesting that it may serve as a relevant nutrient source within the gut niche. Although the pathway lacks synteny with the reference *Pectobacterium* species due to the absence of the D-apiose isomerase, we experimentally confirmed its function via growth assays. Further biochemical studies are warranted to fully characterize the additional enzymes and intermediate compounds in *E. coli*.

Our study has several limitations. First, despite nearly 1,500 genomes, our data set does not encompass the full diversity of *E. coli*. For example, InPEC and *Shigella* strains were not included. The latter, in particular, shows drastic metabolic reduction with convergent evolution in several cases (24, 25), probably due to its host range reduction and intracellular lifestyle. Furthermore, data from remote populations characterized by a very low prevalence of B2 commensal strains (108–111) should be included. Analysis of these strains may further broaden our understanding of *E. coli* metabolic diversity. Second, pathway prediction from genome data can misrepresent metabolic capacities due to over- or under-prediction. Nonetheless, our findings are largely consistent with previous studies, and we functionally validated a novel pathway. Third, we could not assess transcriptional or post-transcriptional metabolic variation, which may obscure functional metabolic differences (112). Last, our binary presence/absence analysis does not consider allelic variation, which could affect enzyme efficiency or affinity and are likely important for niche specialization (6). Such analyses require extensive biochemical data that are not currently available at this scale.

In conclusion, through a large-scale analysis of *E. coli* metabolic pathways in commensals and strains responsible for severe extraintestinal infections, we identified lineage-specific metabolic capacities. While no pathways were strictly associated with pathogenicity or BSI portal of entry, several appeared linked to successful ExPEC clones. These clone-specific metabolic traits may enhance the fitness of major BSI STcs in both gut colonization and extraintestinal infection by providing access to niche-specific nutrient sources (101), thereby allowing them to outcompete resident microbiota and potentially contributing to their emergence and global dissemination. Our findings underscore the strong phylogenetic imprint on *E. coli* metabolism and highlight specific pathways with potential ecological and clinical relevance.

## SUPPLEMENTAL MATERIAL

Supplemental figures jb.00421-25-s0001.pdf Figures S1 to S12.

Supplemental tables jb.00421-25-s0002.xlsx Tables S1 to S9.

ASM does not own the copyrights to Supplemental Material that may be linked to, or accessed through, an article. The authors have granted ASM a non-exclusive, world-wide license to publish the Supplemental Material files. Please contact the corresponding author directly for reuse.
